# Revisiting the Nutritional, Chemical and Biological Potential of *Cajanus cajan* (L.) Millsp.

**DOI:** 10.3390/molecules27206877

**Published:** 2022-10-13

**Authors:** Baby Gargi, Prabhakar Semwal, Shabaaz Begum Jameel Pasha, Pooja Singh, Sakshi Painuli, Ashish Thapliyal, Natália Cruz-Martins

**Affiliations:** 1 Department of Life Sciences, Graphic Era (Deemed to be University), Dehradun 248 002, India; 2 Uttarakhand Council for Biotechnology (UCB), Premnagar, Dehradun 248 006, India; 3 Faculty of Medicine, University of Porto, 4200-319 Porto, Portugal; 4 Institute for Research and Innovation in Health (i3S), University of Porto, 4200-319 Porto, Portugal; 5 Institute of Research and Advanced Training in Health Sciences and Technologies (CESPU), Rua Central de Gandra 1317, 4585-116 Gandra PRD, Portugal; 6 TOXRUN—Toxicology Research Unit, University Institute of Health Sciences, CESPU, CRL, 4585-116 Gandra PRD, Portugal

**Keywords:** legumes, *Cajanus cajan*, bioactive compounds, nutraceuticals, bioactive effects

## Abstract

The genus *Cajanus* (Family: Fabaceae) consists of approximately 37 species, and *Cajanus cajan* (*C. cajan*) is a significant member of the genus. It is a commercial legume crop widely grown in sub-tropical and semi-arid tropical areas of the world. *C. cajan* is well known for its folk medicinal uses to treat various disorders, such as toothache, dizziness, diabetes, stomachache, female ailments and chronic infections. These properties have been linked to the presence of several value-added nutritional and bioactive components. Different solvent extracts from *C.* *cajan* (leaves, root, stem and seeds) have been evaluated for their phytochemical and biological activities, namely antioxidant, antimicrobial, antidiabetic, neuroprotective, and anti-inflammatory effects. Taken together, and considering the prominent nutraceutical and therapeutic properties of *C. cajan,* this review article focuses on the important details including ethnomedicinal uses, chemical composition, biological applications and some other medicinal aspects related to *C.* *cajan* nutraceutical and pharmacological applications.

## 1. Introduction

India contributes significantly to global grain legume production, accounting for approximately 90% of global production and ranking sixth in terms of production and area cultivated [1]. Most legume species belong to the Fabaceae or Leguminosae families and are depicted due to their fruits generally known as pods. Recognized for their great significance as dietary supplement for humans and animals, these legumes, such as pea, cowpea, chickpea, soybean, mung bean, beans, fava beans, lentils, peanut and pigeon pea, have been increasingly investigated for nutraceutical purposes [2].

Grain legumes are often considered as nature’s treasure offered to mankind and are regarded as “poor man’s meat” because of their high quantity of vitamins, minerals, protein (16–50%) and dietary fiber (10–23%) [3]. Moreover, grain legumes also play a crucial role in ecological services, due to their biological nitrogen fixation capacity [4].

*Cajanus cajan* (L.) Millsp. is a leguminous annual woody or perennial plant [5], and the genus *Cajanus* consists of approximately 37 species out of which *C. cajan* is an extensively used commercial legume crop [6]. It is a native genus from ancient Egypt, Africa, Asia and America, and now it has been widely distributed across the tropical and subtropical regions [7]. Globally, *C. cajan* has been recognized by various names, like Pigeon pea (Australia); red gram, tur, arhar, dal (India); mu dou (China); guando (Brazil) [8], and gunga pea, congo pea and non-eye pea in some other parts of the world [9]. Asia is the main producer of pigeon pea, and India alone contributes approximately to 77% of the total area and 90% of the total production around the world [10,11]. Despite the high potential of pigeon pea as a crop, the plant as a whole has been shown to be beneficial for use as food, feed and fuel thanks to its high nutritional value. Thus, the need to implement prior information about *C. cajan* and compile it for convenient access constitutes the main motivation for this work. In this sense, the present study includes all relevant information from the digital platform on the ethnomedicinal uses, bioactive constituents, nutritional value and biological applications of *C. cajan*, also paying attention to aspects related to its geographical distribution and folk consumption.

## 2. Botanical Description

### 2.1. Geographical Distribution and Taxonomy

*C. cajan* is a perennial drought resistance legume commonly cultivated in the sub-tropical and semi-arid tropical areas of the world [12,13]. India is the prime producer, corresponding to approximately 90% of the total global production. It has also been found since ancient times in Africa, Caribbean, Southeast Asia, and Egypt and has been grown at a wide range of altitudes (up to 3000 m) [8]. *C. cajan* is from the Genus *Cajanus*, Family *Fabaceae*, Order *Rosidae*, Class *Magnoliopsida*, and Kingdom *Plantae* [7].

### 2.2. Cytology

The cytological analysis of *C. cajan* showed that it is diploid having 2n = 2x = 22 chromosomes with an average length of 5.73 ± 1.15 µm up to 10.92 ± 2.69 µm and dominantly metacentric in shape, consisting of 14 metacentric and 4 submetacentric chromosomes [14]. *C.* *cajan* has a genome of size 858 mega-base pairs [15]. In the comparative genetic characterization of wild and cultivated *C.* *cajan* genotypes, the cultivated species present maximum polymorphic loci [6].

### 2.3. Morphology

From a morphological point of view, *C.* *cajan* is a short-lived shrub with erect stems of 1–2 m height [16]. Its roots are finely nodulated, lateral and deep rooted of up to 3 m, possessing a root system having a central taproot with several secondary and lateral branches. The branching pattern in *C. cajan* is determined based on the habitat, spacing and plant genotype. The leaves are lanceolate to elliptical in shape and size, ranging from 6 to 17 cm in length and are around the same breadth. The flowers are usually, yellow to orange in color, present a long peduncle of 1–8 cm long and terminal or axillary racemes (4–12 cm). Calyx: gamosepalous with 5 lobes, Corolla: zygomorphic and bright yellow, Androecium: 10 stamens (4 with short filaments and 6 with long filaments), Gynoecium: ovary (superior, pubescent, 2–9 ovules and monocarpellary), style (long, filiform and glabrous), stigma (incurved & thickened), Seeds: spherical or lens shaped [8].

## 3. Traditional Uses

The use of *C. cajan* for traditional purposes dates since immemorial times, and such information has passed over the generations in order to substantially promote the continuity of knowledge improvement. The diversity and availability in regional flora of plant resources is markedly determined by the use of plant species in folk medicinal practices [17]. Various studies have demonstrated that the leaves, seeds, stems and roots of *C. cajan* have been used in traditional medicine for the treatment of various ailments, including toothache, diabetes, dizziness, baldness and gastrointestinal discomfort in few domains of India, Bangladesh, China and many other nations. In Oman, *C. cajan* seeds are used for treating many chronic infections, and native people use the juice from leaves to treat various dermatological conditions [18]. In ancient times, the floral decoction was used for treating pneumonia, coughs, menstrual disorders, dysentery and bronchitis, while leaf decoction was used in Eastern Nigeria for treating measles [19]. A detailed description of the traditional uses of *C. cajan* is presented in Table 1.

## 4. Nutritional Properties

The nutritional profiling of *C. cajan,* including of its leaves, seeds, roots and stem, has also been investigated by standard methods to determine the proximate, amino acid and mineral composition (Table 2). The maximum fat (15.00 ± 0.090%), moisture (8.20 ± 0.229%), carbohydrate (40.95 ± 0.244%) and nutritive value (333.73 ± 1.500%) were recorded in seeds, however the highest protein content was found in leaves (31.99 ± 0.070%) (Table 2). Results of the proximate composition of protein isolate, full fat flour and defatted flour derived from *C. cajan* and its comparisons with wheat flour and yellow-pea flour are shown in Table 3.

The study of amino acids content present in *C. cajan* reveals that leaves (808.8 ± 10.3 mg/100 g) and roots (871.8 ± 11.2 mg/100 g) contain the highest concentration of glutamine, whereas alanine (1547.8 ± 3.9 mg/100 g) and aspartic acid (11.56 g/16 gN) were found in maximum amounts in seeds. The lowest concentration of tryptophan was observed in leaves (2.4 ± 0.4 mg/100 g), roots (1.3 ± 0.4 mg/100 g) and seeds (9.5 ± 0.1 mg/100 g). The detail description of the amino acid composition is mentioned in Table 4.

To what concerns to mineral composition, the evaluation of *C. cajan* revealed higher levels of calcium in leaves (33 ± 4.9 mg/100 g), seeds (581 ± 4.3 mg/100 g) and roots (597 ± 2.5 mg/100 g) and lower levels of zinc (2.1 ± 0.9, 0.7 ± 0.2 and 0.7 ± 0.9 mg/100 g, respectively) (Table 5). Due to the nutritional contribution and health benefits of *C. cajan* it is regarded as an alternative to produce vegetable meat, with high quality standards and appropriate sensory characteristics that allow consumer acceptance and integration of product in daily diet. Moreover, due to its essential nutrient content, this makes an exquisite preference for vegetarian consumers.

Scientific investigations of nutraceutical profiling have underlined that *C. cajan* has relevant nutritional attributes that help in the treatment of different types of human conditions.

## 5. Chemical Composition

**Figure 1:**
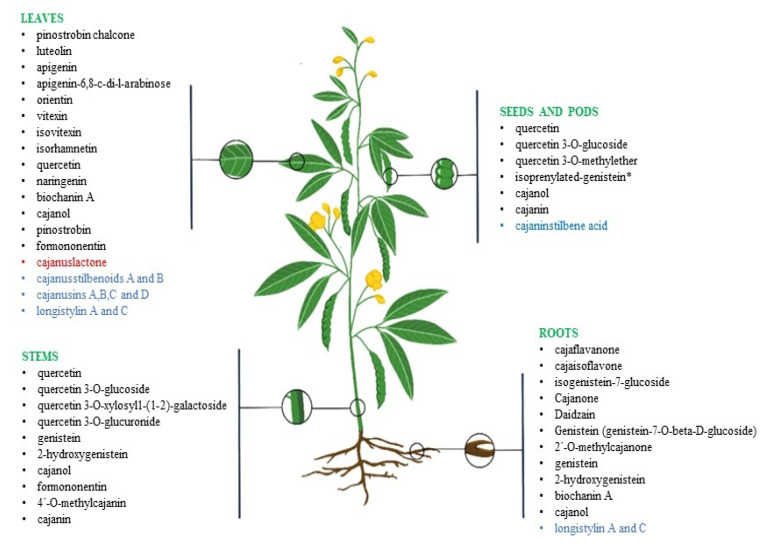
Localization of bioactive components (flavonoids—black, stilbenoids—blue, coumarin—red) in different parts of *Cajanus cajan.* Note—“*” Isoprenylated-genistein detected in seedlings only.

The composition and concentration of active compounds presents in plant matrices largely determines their bioactive effects. In *C. cajan* the main bioactive compounds identified to date are broadly classified into the flavonoids, phenolics and stilbenes group [36,37]. The literature-based screening of phytochemicals revealed the presence of various phenolic compounds, namely, cajanol, longistylin A and C, genistein and biochanin A [38]. The total phenolic content of *C. cajan* seeds, root and stem was estimated to be between 4.27–92.00 mg of gallic acid equivalent (GAE) per gram dry weight (DW) (mg GAE/g DW) extract by using different solvent systems (dichloromethane, water and methanol) [38]. The determination of the chemical composition of ethanol leaves extract by high-performance liquid chromatography (HPLC) analysis revealed the presence of seven flavonoids, including pinostrobin, orientin, naringenin, apigenin, apigenin-6,8-di-C-α-L-arabinopyranoside and pinostrobin chalcone, and two stilbenes, namely cajaninstilbene acid and longistyline C [36]. In a study, Zhang and colleagues [39] reported the structure of a novel prenylated flavonone isolated from *C. cajan*, naringenin-3’-isoprenyl-7-methyl ether 1, by 1D and 2D NMR technology. Other phytochemical studies also indicated the existence of acidic compounds, glycosides, tannins, resins, saponins and reducing sugars [40,41,42]. The description of the bioactive components present in different parts of *C. cajan* is shown in Figure 1 and Table 6.

Looking at the essential oil from *C. cajan*, Ogunbinu et al. [44] identified the presence of 100 constituents in seeds, stem and leaves using gas chromatography (GC) and gas chromatography-mass spectrometry (GC-MS) analysis. Among all compounds, sesquiterpene hydrocarbons were found in higher amounts in 81.2% (stem), 92.5% (leaves), and 94.3% (seeds). Esters, aldehydes, alcohols, terpenoids and ketones and some other constituents, including α-himachalene, β- himachalene, γ-himachalane, α-humulene and α-copaene were also identified. Qi et al. [45] reported the presence of 27 compounds in the essential oil from *C. cajan* leaves extracted through solvent-free microwave extraction (SFME) and hydro-distillation (HD) methods. Sesquiterpenes were the most abundant compounds identified, namely α-humulene, α-copaene, α-bisabolene, α-himachalene, β-caryophyllene and alloaromadendrene. The details of other constituents are listed in Table 7.

## 6. Biological Applications

**Figure 2:**
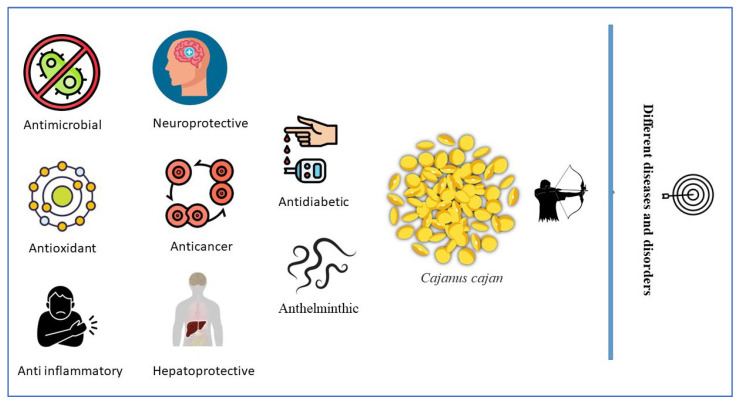
Different biological applications of *Cajanus cajan*.

With the growth of world’s economy and enhancement in people’s living standard, several chronic diseases, like neurological, metabolic, inflammatory, cerebrovascular and cardiovascular disorders have increased rapidly [46]. Natural products are widely recognized for their biological or pharmacological potential since ancient times, and recently the interest in their study has re-emerged as upcoming drug candidates. Globally, around 50,000 plants have shown potent therapeutic potentialities [47]. According to pharmacological studies, *C. cajan* leaves have various bioactivities, including antioxidant, antiplasmodial, anticancer, hypoglycemic, insecticidal, neuroprotective and antimicrobial activities [37,48]. Moreover, the molecular regulatory mechanism of few biological applications/activity are briefly summarized in Table 8. The most relevant therapeutic applications of *C. cajan* briefly described below and presented in Figure 2.

### 6.1. Antimicrobial Activity

The antimicrobial activity of plants varies pronouncedly depending on chemical constituents presents, hence it is difficult to classify single antimicrobial mechanisms, as they rely on the phytochemical properties of the plant [57]. Dinore and Farooqui (2022) [58] investigated the antimicrobial activity of *C. cajan* leaves methanol extract against *Escherichia coli* and *Candida albicans*, and the results indicated a remarkable ability to inhibit the growth of the microorganisms, with minimum inhibitory concentrations (MIC) of 50 µg/mL and minimum fungicidal concentrations (MFC) of 250 µg/mL. Cajanuslactone, one of the most abundant phytoconstituents present in *C. cajan* leaves is expected to be the responsible for the marked antimicrobial properties [22]. The antifungal potential of *C. cajan* roots were examined by microdilution method to demonstrate the use of plant extract as a novel therapeutic source [59]. The ethanolic extract of the roots showed antifungal activity in terms of MIC (*Candida albicans* 512 µg/mL, *Candida krusei* 512 µg/mL and *Candida tropicalis* 512 µg/mL) [59].

In another study, Qi et al. [45] extracted the essential oil from *C. cajan* leaves by solvent free microwave extraction and reported antimicrobial properties to the extracted oil. The essential oil revealed an effective antimicrobial potential, addressed through determination of MIC and minimum bactericidal concentration (MBC), against *Bacillus subtilis* (1.06 and 2.12 mg/mL, respectively), and *Propionibacterium acnes* (0.13 and 0.26 mg/mL, respectively). Additional studies on antimicrobial potential of *C. cajan* (different parts) are listed in Table 9 and Table 10.

### 6.2. Antioxidant Activity

Different studies also have been performed to assess the antioxidant potential of different parts of *C. cajan*. Aggarwal et al. (2015) reported the antioxidant potential of *C. cajan* ethanol seed extract using ferric reducing antioxidant power (FRAP) assay. The results obtained revealed a concentration-dependent antioxidant activity (concentration 25 to 450 µg, 4.4 to 43.0 µM) [64].

The HPLC-FRAP analysis of *C. cajan* stem bark extract, revealed that it consists of 12 phenolic compounds with notable antioxidant activity [52]. Yang et al. (2020) performed DPPH (2,2-diphenyl-1-picrylhydrazyl), NO (Nitric Oxide) scavenging, ABTS (2,2′-azino-bis(3-ethylbenzothiazoline-6-sulfonic acid) and FRAP (Ferric reducing antioxidant power) assays for determining the antioxidant potential of leaves, seeds and roots of *C. cajan*. Among them, *C. cajan* roots showed high antioxidant efficiency than seeds and leaves [30]. Further data on the antioxidant potential of *C. cajan* and the respective assays are shown in Table 11.

### 6.3. Anti-Diabetic Activity

The antidiabetic potential of *C. cajan* methanol root extract was addressed by Nahar et al. (2014) in alloxan-induced diabetic Swiss albino mice. The experimental mice were treated with *C. cajan* extract up to 5 days (200 and 400 mg/kg bw, orally). Glucose tolerance test and hyperglycemic effect studies (involving diabetes induction in mixed sex Swiss albino mice by injection of aqueous alloxan monohydrate, 55 mg/kg, intravenously) were carried out on tested animals, along with determination of the antioxidant activity. The results showed a rapid decline in fasting serum glucose level (*p* < 0.001) and blood glucose level (*p* < 0.001) in 5 days. On the basis of these results, the plant extract evidenced potent hypoglycemic and antioxidant properties compared to other species (e.g., *Tamarindus indica* seeds) [65].

### 6.4. Tyrosinase Inhibitory Activity

*C. cajan* root, stems and seeds were also addressed for its ability to inhibit tyrosinase activity, and for that water, dichloromethane and methanol extracts were prepared. The IC_50_ values of the extracts varied from 3.55–12.43 mg/mL, whereas the maximum inhibitory capacity was reported for methanol root extract (IC_50_ = 3.55 mg/mL) [38].

### 6.5. Neuroprotective Activity

A variety of naturally-occurring bioactive compounds are currently being explored for their therapeutic potential in neurodegenerative diseases, but only a few are known to have benefits [68]. The use of plant extracts and their bioactive constituents are one of the promising approaches for the treatment of neurological diseases [69]. *C. cajan* was also exploited for their neuroprotective abilities. The presence of stilbenoids is able to induce apoptotic neuronal death by Aβ_25–35_ injection in mice and cause elevation in choline acetyltransferase (ChAT) and superoxide dismutase (SOD) activity in the cortex and hippocampus [70]. In a study with injured larvae of zebrafish, cajanin stilbene acid (CSA) and its derivative were found to decline the migration and production of primitive macrophages and neutrophiles [71], being thus proposed that *C. cajan* may be a promissory source of biomolecules with neuroprotective abilities.

### 6.6. Other Bioactivities

In addition to the above listed bioactive effects of *C. cajan,* other bioactivities, such as hepatoprotective [26,72,73,74,75], anthelminthic [76], anticancer [77], and anti-inflammatory [78] effects have been documented by other researches. Moreover, the *C. cajan* is also used in paper-making, cosmetic industries and multi-purposely in dietary supplements for human and animals.

## 7. Conclusions and Future Prospects

Pigeon pea (*C. cajan*) is one of the most commonly and widely used, tropical and subtropical legume due to its nutrient packed edible seeds, might being effectively used for food and medicinal purposes. However, it is an underutilized/neglected legume species. As yet, several flavonoids, isoflavonoids, tannins, phenolics and proteins have been isolated from various plant parts, and their therapeutic properties have also been confirmed, but many pure and bioactive components were still not taken into consideration. Several studies have identified that the phytochemicals present display excellent bioactive effects for a plethora of human conditions.

A number of extensive research has been done only on extracts rather than isolated fractions and oils, that indicates necessity of further study in this direction. Moreover, majority of studies are limited to in vitro screening, with only a few focusing on *in vivo* testing. As a result, advanced research is required to explore new phytopharmaceuticals based on *C. cajan*. Clinical trials should be conducted to assess the toxicity profile of *C. cajan* in humans in respect of antioxidant activity, antimicrobial activity, anthelminthic activity, anti-inflammatory activity, antidiabetic activity and immunomodulatory aspects. The current review article aims to concentrate attention of researchers as well as pharmaceutical industries on untouched and unexplored aspects related to *C. cajan* and may serve as a crucial link towards the establishment of *C. cajan* as a therapeutic drug. Although, as it is a leguminous plant and plays a major role in biological nitrogen fixation, further more relevant knowledge regarding the characteristics of soil, indigenous microbes and plant species-specific responses is required to establish the inoculant for maximum ecological restoration benefits and to support future adoption of this practice.

## Figures and Tables

**Table 1:** Ethnomedicinal uses of *Cajanus cajan* from different regions.

Medicinal Use	Plant Part	Region	References
Gastrointestinal disorders	Seeds (O)	Trinidad and Tobago	[20]
Menstrual problems	Seeds (O)	India	[21]
Toothache	Stem (T)	China	[22]
Sedative	Seeds (O)	India	[21]
Wounds	Stem (T)	Nigeria	[23]
Diabetes	Seeds, Leaves (O)	Bangladesh, India	[22,24]
Laxative	Leaves (O)	India, India, China	[25,26,27]
Dizziness	Seeds (O)	India	[20]
Poultice	Seeds (T)	India	[28]
Wormicide	Seeds, Roots (T)	India	[20]
Baldness	Seeds (T)	India	[28]
Gingivitis, Stomatitis, Toothbrush	Stem, Seeds, Leaves (T)	India, China, Thailand	[18,23,27]
Genital inflammations	Leaves (T)	India	[20]
Malaria	Leaves (O)	Nigeria	[23]
Ulcers	Leaves (T)	India	[25]
Syphilis	Roots (O)	India	[20]
Cough	Roots (O)	India	[20]
Measles	Seeds (T)	China	[22]
Energy stimulant	Seeds (O)	Bangladesh	[24]
Induce lactation	Leaves and Seeds (T)	India	[27]
Nullify effect of intoxication	Leaves (O)	India	[27]

(O) = Oral; (T) = Topical.

**Table 2:** Proximate composition of *Cajanus cajan* from different countries.

Proximate	Seeds (%) [5] (Nigeria)	Seeds (%) [29] (India)	Seeds (%) [30] (Taiwan)	Seeds (%) [31] (India)	Leaves (%) [32] (Nigeria)	Leaves (%) [29] (India)	Leaves (%) [30] (Taiwan)	Roots (%) [30] (Taiwan)	Stem (%) [29] (India)	Seeds (%) [33] (India)
Dry matter	95.89	91.80 ± 0.22	-	-	-	93.68 ± 0.284	-	-	93.88 ± 0.12	-
Protein	21.03	08.62 ± 0.03	22.0 ± 0.4	25.46	22.40	31.99 ± 0.070	19.4 ± 0.5	2.4 ± 0.1	21.34 ± 0.56	19.53 ± 0.02
Fat	4.43	15.00 ± 0.09	5.5 ± 0.3	1.65	2.74	13.00 ± 0.090	ND	0.4 ± 0.0	14.19 ± 0.26	1.64 ± 0.03
Fibre	7.16	05.09 ± 0.08	-	6.50	7.25	21.82 ± 0.238	-	-	27.70 ± 0.36	4.75 ± 0.02
Ash	3.76	22.11 ± 0.11	12.0 ± 0.0	3.66	8.22	20.60 ± 0.114	3.6 ± 0.1	3.6 ± 0.2	23.00 ± 0.22	3.23 ± 0.03
Moisture	-	8.20 ± 0.22	14.3 ± 0.1	8.50	11.20	06.31 ± 0.284	11.5 ± 0.2	3.3 ± 0.1	06.11 ± 0.12	8.17 ± 0.02
Carbohydrate	-	40.95 ± 0.24	56.2 ± 0.3	54.23	-	6.269 ± 0.153	65.6 ± 0.2	90.3 ± 0.1	8.131 ± 0.38	62.28 ± 0.05
Nutritive value	-	333.73 ± 1.50	-	-	-	236.72 ± 0.591	-	-	242.61 ± 1.56	-

ND = Not detectable; “-” = Not tested.

**Table 3:** Comparison of *Cajanus cajan* flour proximate composition with generally used flours.

Proximate	Full Fat Flour (*Cajanus cajan*) [34]	Defatted Flour (*Cajanus cajan*) [34]	Protein Isolate (*Cajanus cajan*) [34]	Wheat Flour (*Triticum aestivum)* [35]	Yellow-Pea Flour (*Pisum sativum*) [35]
Protein	24.02 ± 0.016%	26.30 ± 0.016%	90.65 ± 0.025%	12.81 ± 0.06%	22.33 ± 0.05%
Moisture	6.85 ± 0.012%	6.76 ± 0.016%	6.63 ± 0.015%	12.70 ± 0.0%	13.35 ± 0.1%
Fibre	1.24 ± 0.016%	1.56 ± 0.015%	-	10.08 ± 1.20%	14.84 ± 0.93%
Fat	2.017 ± 0.062%	-	-	1.53 ± 0.08%	1.40 ± 0.04%

“-” = Not tested.

**Table 4:** Amino acid composition of different parts of *Cajanus cajan*.

Amino Acids	Seeds (mg/100 g) [30]	Leaves (mg/100 g) [30]	Roots (mg/100 g) [30]
Lysine	740.8 ± 6.3	425.4 ± 10.1	297.9 ± 2.0
Histidine	361.7 ± 3.6	266.8 ± 1.3	118.4 ± 4.3
Arginine	279.9 ± 2.6	333.4 ± 1.3	226.1 ± 5.9
Aspartic acid	126.4 ± 1.7	323.3 ± 5.2	112.5 ± 5.2
Threonine	136.2 ± 5.4	406.8 ± 1.3	119.9 ± 4.6
Serine	220.0 ± 8.1	494.6 ± 4.8	169.2 ± 4.5
Glutamic acid	-	-	-
Proline	72.1 ± 8.2	137.9 ± 1.2	89.1 ± 8.1
Glycine	160.7 ± 3.4	235.7 ± 2.8	139.7 ± 6.9
Alanine	1547.8 ± 3.9	576.5 ± 5.6	687.5 ± 12.3
Cystine	ND	ND	ND
Valine	671.4 ± 4.8	422.2 ± 3.6	381.1 ± 5.6
Methionine	70.6 ± 1.6	86.0 ± 2.3	61.1 ± 1.2
Isoleucine	392.0 ± 3.1	314.1 ± 8.3	272.7 ± 4.2
Leucine	679.7 ± 13.5	597.8 ± 3.8	492.2 ± 4.2
Tyrosine	186.1 ± 2.0	143.9 ± 3.9	149.8 ± 5.2
Phenylalanine	354.7 ± 7.6	612.4 ± 3.6	262.1 ± 2.5
Tryptophan	9.5 ± 0.1	2.4 ± 0.4	1.3 ± 0.4
Glutamine	648.3 ± 6.3	808.8 ± 10.3	871.8 ± 11.2

ND = Not detectable; “-” = Not tested.

**Table 5:** Mineral composition of *Cajanus cajan*.

Minerals	Seeds (mg/100 g) [30] (Taiwan)	Seeds (mg/100 g) [8] (India)	Seeds (mg/100 g) [33] (India)	Leaves (mg/100 g) [30] (Taiwan)	Roots (mg/100 g) [30] (Taiwan)
Sodium (Na)	32.5 ± 5.5	-	-	19.7 ± 39.0	108.0 ± 7.6
Zinc (Zn)	0.7 ± 0.2	2.3	0.585 ± 0.04	2.1 ± 0.9	0.7 ± 0.9
Magnesium (Mg)	138.8 ± 7.2	122.0	-	111 ± 9.5	130 ± 8.7
Manganese (Mn)	6.8 ± 5.2	-	0.204 ± 0.04	ND	0.7 ± 0.2
Iron (Fe)	51.5 ± 8.7	3.9	0.335 ± 0.08	4.8 ± 2.1	ND
Copper (Cu)	1.4 ± 0.6	1.3	0.052 ± 0.03	ND	1.0 ± 0.6
Calcium (Ca)	581 ± 1.3	120.8	-	33 ± 4.9	597 ± 2.5

ND = Not detectable; “-” = Not tested.

**Table 6:** Bioactive components present in *Cajanus cajan* from different regions.

Bioactive Compound	Part Used	Extract	Region	Ref.
Cajanuslactone	Leaves	Chloroform	India	[26]
Cajanin, Longistylin C, Longistylin A, Betulinic acid, Pinostrobin, Cajaninstilbene acid, Orientin, Vitexin	Leaves	Ethanol	India	[26]
Protein fraction Cl-1	Leaves	Methanol	India	[26]
Genistein, Genistin	Roots	Ethanol: Water	India	[26]
Cajanol (isoflavonoids)	Roots	Ethanol	India	[26]
Phenolics (flavonoids, tannis)	Aerial plants	Hydroalcoholic	China	[43]
Cajaninstilbene acid, Vitexin, Orientin, Pinostrobin	Leaves	Ethanol	Bangladesh	[7]
Luteolin, Apigenin, Quercitin, Isorhamnetin, Cajaninstilbene acid, Pinostrobin, Cajanin, Longistylin A, Longistylin C	Leaves	-	Bangladesh	[7]
Cajanuslactone	Leaves	Chloroform	Bangladesh	[7]
Hordenine, Juliflorine, Betulinic acid, Stigmasterol, Beta-sitosterol	Leaves	-	Bangladesh	[7]

**Table 7:** Essential oil composition of *Cajanus cajan*.

Plant Part	Bioactive Compounds	Region	Ref.
Leaves	(E)-2-Hexenal; Benzaldehyde; Nonanal; Decanal; α-Longipinene; Cyclosativene; α-Copaene; β-Longipinene; (Z)-Caryophyllene; Longifolene; α-Gurjunene; trans−α-Bergamotene; α-Guaiene; α-Himachalene; α-Humulene; allo-Aromadendrene; γ-Muurolene; γ-Himachalene; β-Selinene; α-Selinene; β-Himachalene; β-Bisabolene; trans-γ-Cadinene; α-Dehydro-ar-himachalene; δ-Cadinene; trans-Calamenene; γ-Dehydro-ar-himachalene; trans-Cadina-1(2),4-diene; α-Calacorene; trans-Nerolidol; Ledol; Caryophyllenyl alcohol; Himachalene epoxide; Caryophyllene oxide; Globulol; Longiborneol (=juniperol); Humulene oxide II; β-Himachalene oxide; Bisabolol-11-ol; 1-epi-Cubenol; α-Acorenol; τ-Cadinol; τ-Muurolol; α-Muurolol; Himachalol; Selin-11-en-4α-ol; β-Bisabolol; Cadalene; α-Bisabolol; epi-α-Bisabolol; Hexahydrofarnesyl acetone; ar-Himachalene-2-ol; n-Docosane.	Nigeria	[44]
Stem	n-Decane; Limonene; Nonanal; Citronellal; 1-Nonanol; Menthol; Methyl salicylate; Decanal; α-Longipinene; Cyclosativene; α-Copaene; n-Tetradecane; Longifolene; Dodecanal; β-Caryophyllene; trans−α-Bergamotene; α-Guaiene; α-Himachalene; α-Humulene; allo-Aromadendrene; γ-Muurolene; γ-Himachalene; β-Selinene; Bicyclosesquiphellandrene (=trans-muurola-4(14),5 diene); α-Selinene; β-Himachalene; β-Bisabolene; trans-γ-Cadinene; α-Dehydro-ar-himachalene; δ-Cadinene; trans-Calamenene; γ-Dehydro-ar-himachalene; α-Cadinene; α-Calacorene; Germacrene B; trans-Nerolidol; Caryophyllenyl alcohol; Himachalene epoxide; Caryophyllene oxide; Longiborneol (=juniperol); Humulene oxide II; β-Himachalene oxide; 1-epi-Cubenol; α-Acorenol; cis-Cadina-4-en-7-ol; τ-Cadinol; Cubenol; α-Muurolol; Himachalol; α-Cadinol; n-Octadecane; Hexahydrofarnesyl acetone; n-Docosane.	Nigeria	[44]
Seeds	Benzaldehyde; Nonanal; α-Longipinene; Cyclosativene; α-Copaene; (Z)-Caryophyllene; Longifolene; α-Gurjunene; β-Caryophyllene; β-Cedrene; β-Duprezianene; β-Gurjunene; trans−α-Bergamotene; α-Guaiene; α-Himachalene; α-Humulene; allo-Aromadendrene; γ-Muurolene; γ-Himachalene; β-Selinene; α-Selinene; β-Himachalene; β-Bisabolene; δ-Cadinene; γ-Dehydro-ar-himachalene; trans-Cadina-1(2),4-diene; 10-epi-Cubebol; α-Cadinene; α-Calacorene; trans-Nerolidol; Caryophyllenyl alcohol; Himachalene epoxide; Caryophyllene oxide; Globulol; Viridiflorol; Longiborneol (=juniperol); Humulene oxide II; β-Himachalene oxide; Bisabolol-11-ol; epi-10-γ-Eudesmol; 1-epi-Cubenol; cis-Cadina-4-en-7-ol; τ-Cadinol; τ-Muurolol; α-Muurolol; Himachalol; Selin-11-en-4α-ol; Bulnesol; β-Bisabolol; Cadalene; α-Bisabolol; epi-α-Bisabolol; Hexahydrofarnesyl acetone.	Nigeria	[44]
Leaves	3,6-Dimethyl-octane; Naphthalene; Dodecane; 6-Ethyl-undecane; 4-Methyl-Dodecane; 4-Ethy-undecane; 4,6-Dimethyl-dodecane; 1-Methyl-naphathalene; 2,6,11-Trimethyl-dodecane; α-Longipinene; 2-Methyl-tridecane; (+)-Cyclosativene; α-Copaene; Tetradecane; Longifolene; Caryophyllene; α-Selinene; β-Bergamotene; α-Himachalene; Humulene; Alloaromadendrene; α-Bisabolene; 2,4-Bis(1,1-dimethylethyl)-phenol; Hexadecane; Norphytane.	China	[45]

**Table 8:** Molecular regulatory mechanism of few biological activities of *Cajanus cajan*.

S. No.	Biological Activity	Isolated Compounds/Extracts	Biological Activity	Reference
1	Hypocholesterolemic	Methanolic extract	↑ LDRL; ↓PCSK9 mRNA	[49]
2	Antidepressant	Cajanin stilbene acid	↓ Kynurenine pathway	[50]
3	Neuroprotective	AgNP	↑ Proline; ↑ Glyoxalase; ↑ Pyrroline-5-carboxylate synthetase gene.	[51]
4	Antidepressant	Fluoride exposure	↓ Growth and membrane stability index; ↑ Reactive oxygen species; ↑ Malondialdehyde; ↑ Glutathione; ↑ Lipoxygenase.	[51]
5	Antioxidant	Hexane extract	↓ AChE; ↓ BChE; ↓ α-AMYLASE; ↓ α-glucosidase.	[52]
6	Antidiabetic	Methanolic extract	↓ Fasting blood sugar	[53]
7	Anticancer	Betulinic acid, genistin, orientin and vitexin	↓ Inhibit the Histone deacetylases enzyme	[54]
8	Antimitotic	-	↓ Decrease the percentage of Sea urchin embryonic cells	[55]
9	Anticancer	Cajanin stilbene acid	↓ Inhibit several human kinases, ↓ serine/threonine-protein kinase WNK3	[56]

LDRL = Low density lipoprotein receptor; PCSK9 = Proprotein convertase subtilisin/kesin type 9; AgNP = Silver nanoparticles; AChE = Acetyl cholinesterase; BChE = Butyryl cholinesterase; ↑: Increased; ↓: Decreased.

**Table 9:** Antibacterial activity of *Cajanus cajan*.

Plant Part	Solvent System	Concentration of Extract	Microorganism	Agar Well Diffusion Method/ Agar Disc Diffusion Method		Region	Ref.
				ZI (mm)	MIC (mg/mL)		
Leaves	Methanol	6.25–200.00 µg/mL	*Escherichia coli* *Staphylococcus aureus* *Bacillus subtilis* *Salmonella typhi*	4.80–11.69 0.66–8.52 0.07–1.92 -	6.25 50.00 200.00 -	Nigeria	[60]
Leaves	Ethanol	6.25–200.00 µg/mL	*Escherichia coli* *Staphylococcus aureus* *Bacillus subtilis* *Salmonella typhi*	6.31–11.90 2.28–7.46 1.08 4.00–9.85	3.13 100.00 400.00 12.50	Nigeria	[60]
Leaves	Acetone	6.25–200.00 µg/mL	*Escherichia coli* *Staphylococcus aureus* *Bacillus subtilis* *Salmonella typhi*	1.80–10.25 2.10–8.55 1.23 6.19–8.46	12.50 50.00 400.00 3.13	Nigeria	[60]
Leaves	Hot water	6.25–200.00 µg/mL	*Escherichia coli* *Staphylococcus aureus* *Bacillus subtilis* *Salmonella typhi*	1.04 - - 1.04–2.09	800.00 - - 200.00	Nigeria	[60]
Leaves	Cold water	6.25–200.00 µg/mL	*Escherichia coli* *Staphylococcus aureus* *Bacillus subtilis* *Salmonella typhi*	- - - 3.69	- - - 200.00	Nigeria	[60]
Leaves	Petroleum ether	12.5–100 mg/mL	*Bacillus subtilis* *Staphylococcus aureus* *Streptococcus pneumoniae* *Salmonella typhi* *Klebsiella pneumoniae* *Escherichia coli* *Pseudomonas aeruginosa*	11.5–13.0 10.0–11.0 08.0–11.5 11.0–11.5 10.0–13.5 07.1–12.1 -	6.25 60 - 70 65 12.5 -	India	[61]
Leaves	Chloroform	12.5–100 mg/mL	*Bacillus subtilis* *Staphylococcus aureus* *Streptococcus pneumoniae* *Salmonella typhi* *Klebsiella pneumoniae* *Escherichia coli* *Pseudomonas aeruginosa*	12.6–18.1 12.6–15.0 11.6–14.0 - - - -	6.25 75.00 - - - - -	India	[61]
Leaves	Methanol	12.5–100 mg/mL	*Bacillus subtilis* *Staphylococcus aureus* *Streptococcus pneumoniae* *Salmonella typhi* *Klebsiella pneumoniae* *Escherichia coli* *Pseudomonas aeruginosa*	14.0–19.0 15.0–20.0 12.0–17.0 08.0–12.0 18.4–25.7 10.4–16.0 -	6.25 6.25 - 6.25 3.125 12.50 -	India	[61]
Leaves	Ethanol	12.5–100 mg/mL	*Bacillus subtilis* *Staphylococcus aureus* *Streptococcus pneumoniae* *Salmonella typhi* *Klebsiella pneumoniae* *Escherichia coli* *Pseudomonas aeruginosa*	16.0–22.1 17.1–30.1 14.1–22.1 11.0–18.0 22.0–25.5 12.3–17.1 10.5–13.4	6.25 3.125 - 6.25 70.00 6.25 50.00	India	[61]
Leaves	Aqueous	12.5–100 mg/mL	*Bacillus subtilis* *Staphylococcus aureus* *Streptococcus pneumoniae* *Salmonella typhi* *Klebsiella pneumoniae* *Escherichia coli* *Pseudomonas aeruginosa*	12.5–18.5 11.0–16.3 10.0–16.0 10.0–15.1 16.4–17.2 13.6–16.6 -	6.52 6.25 - 6.25 6.25 6.25 -	India	[61]
Leaves	Methanol	40 mg/mL	*Pseudomonas aeruginosa*	13	-	Nigeria	[62]

ZI: Zone of inhibition; MIC: Minimum inhibitory concentration; “-”: No zone of inhibition and MIC.

**Table 10:** Antifungal activity of *Cajanus cajan*.

Plant Part	Solvent System	Extract Concentration (mg/mL)	Microorganism	Agar Well Diffusion Method/ Agar Disc Diffusion Method		Region	Ref.
				ZI (mm)	MIC (mg/mL)		
Leaves	Methanol	3.15–50	*Aspergillus niger* *Candida albicans*	16–17 17–18	10–13 10–14	Sudan	[63]
Leaves	Ethanol	3.15–50	*Aspergillus niger* *Candida albicans*	- 14–15	- -	Sudan	[63]
Leaves	Petroleum ether	3.15–50	*Aspergillus niger* *Candida albicans*	- -	- -	Sudan	[63]
Leaves	Ethyl acetate	3.15–50	*Aspergillus niger* *Candida albicans*	- -	- -	Sudan	[63]
Leaves	Chloroform	3.15–50	*Aspergillus niger* *Candida albicans*	- -	- -	Sudan	[63]
Leaves	Methanol	12.5–200	*Basidiobolus species* *Trichophyton rubrum* *Trichophyton mentagrophtye*	- 2 -	- 1 -	Nigeria	[42]
Roots	Ethanol	0.1	*Candida albicans* *Candida krusei* *Candida tropicalis*	- - -	0.512 0.512 0.512	Brazil	[59]

ZI: Zone of inhibition; MIC: Minimum inhibitory concentration; “-”: No zone of inhibition and MIC.

**Table 11:** Antioxidant potential of *Cajanus cajan*.

Part Used	Solvent System	Experiment/Assay	Antioxidant Potential	Ref.
Leaves	Aqueous	Free radical scavenging (DPPH) Ferric reducing antioxidant power (FRAP) Hydroxyl radical scavenging (OH)	IC_50_ = 0.69 mg/mL IC_50_ = 115.9 mg/mL IC_50_ = 2.4 µg/mL	[1]
Leaves	Ethanol	Free radical scavenging (DPPH) Ferric reducing antioxidant power (FRAP) Hydroxyl radical scavenging (OH)	IC_50_ = 0.79 mg/mL IC_50_ = 145.8 mg/mL IC_50_ = 2.6 µg/mL	[1]
Root	Methanol	Free radical scavenging (DPPH)	IC_50_ = 17.44 µg/mL	[65]
Leaves	Ethanol	Free radical scavenging (DPPH) β-carotene-linoleic acid test	IC_50_ = 242.01 µg/mL IC_50_ = 256.88 µg/mL	[43]
Leaves	Aqueous	Free radical scavenging (DPPH) β-carotene-linoleic acid test	IC_50_ = 404.91 µg/mL IC_50_ = 475.26 µg/mL	[43]
Seeds	Methanol	ABTS assay Ferric reducing antioxidant power (FRAP)	109.07 ± 0.2 49.08 ± 0.5 µM/mL	[66]
Seeds	Aqueous	ABTS scavenging assay Ferric reducing antioxidant power (FRAP)	140.69 ± 0.3 44.08 ± 0.1	[66]
Seeds	Methanol	Free radical scavenging (DPPH) Metal chelating (Fe^2+^) activity Ferric reducing antioxidant power (FRAP)	21.57 ± 0.49% 42.02 ± 1.11% 98.93 ± 1.89 µg AAE/g	[67]
Root	Hot water	Free radical scavenging (DPPH) Nitric Oxide (NO) scavenging effects assay ABTS scavenging assay	736 ± 15 µg/mL 145 ± 6 µg/mL 477 ± 89 µg/mL	[30]
Seeds	Hot water	Free radical scavenging (DPPH) Nitric Oxide (NO) scavenging effects assay	2536 ± 51 µg/mL 1250 ± 23 µg/mL	[30]
Leaves	Hot water	Free radical scavenging (DPPH) Nitric Oxide (NO) scavenging effects assay	752 ± 12 µg/mL 650 ± 20 µg/mL	[30]
Root	Ethanol	Free radical scavenging (DPPH) Nitric Oxide (NO) scavenging effects assay	640 ± 16 µg/mL 51 ± 4 µg/mL	[30]
Seeds	Ethanol	Free radical scavenging (DPPH) Nitric Oxide (NO) scavenging effects assay	1263 ± 31 µg/mL 512 ± 16 µg/mL	[30]
Leaves	Ethanol	Free radical scavenging (DPPH) Nitric Oxide (NO) scavenging effects assay	675 ± 13 µg/mL 217 ± 12 µg/mL	[30]
Stem bark	Hexane	Free radical scavenging (DPPH) ABTS scavenging assay Cupric ion reducing antioxidant capacity Ferric reducing antioxidant power (FRAP) Total antioxidant capacity Metal chelating ability (Fe^+2^)	- 4.75 ± 0.32 mg TE/g 13.48 ± 0.30 mg TE/g 8.54 ± 0.19 mg TE/g 0.31 ± 0.02 mg TE/g 6.52 ± 0.29 mg TE/g	[52]
Stem bark	Ethyl acetate	Free radical scavenging (DPPH) ABTS^+^ scavenging assay Cupric ion reducing antioxidant capacity Ferric reducing antioxidant power (FRAP) Total antioxidant capacity Metal chelating ability (Fe^+2^)	12.39 ± 0.09 mg TE/g 22.45 ± 0.70 mg TE/g 72.09 ± 1.40 mg TE/g 25.91 ± 0.44 mg TE/g 1.68 ± 0.09 mg TE/g 10.24 ± 0.03 mg TE/g	[52]
Stem bark	Methanol	Free radical scavenging (DPPH) ABTS scavenging assay Cupric ion reducing antioxidant capacity Ferric reducing antioxidant power (FRAP) Total antioxidant capacity Metal chelating ability (Fe^+2^)	38.41 ± 0.05 mg TE/g 70.49 ± 3.62 mg TE/g 81.86 ± 2.40 mg TE/g 42.96 ± 0.59 mg TE/g 1.32 ± 0.06 mg TE/g 17.00 ± 1.26 mg TE/g	[52]
Stem bark	Infusion	Free radical scavenging (DPPH) ABTS scavenging assay Cupric ion reducing antioxidant capacity Ferric reducing antioxidant power (FRAP) Total antioxidant capacity Metal chelating ability (Fe^+2^)	25.84 ± 0.64 mg TE/g 64.40 ± 1.09 mg TE/g 53.51 ± 0.34 mg TE/g 36.43 ± 0.08 mg TE/g 1.00 ± 0.05 mg TE/g 10.16 ± 0.69 mg TE/g	[52]

DPPH = 2,2-diphenyl-1-picrylhydrazyl; ABTS = 2,2-azino-bis (3-ethylbenzothiazoline-6-sulfonic acid); AAE = Ascorbic acid equivalent; TE = Trolox equivalent.

## Data Availability

Not applicable.
